# Synergistic Regenerative Strategies: Combining Polydeoxyribonucleotide with Biochemical and Physical Agents

**DOI:** 10.3390/ijms27104355

**Published:** 2026-05-14

**Authors:** Jaeseok Choi, Su Kil Jang, Deugchan Lee, Yeong-Min Yoo

**Affiliations:** 1 Institute of Environmental Research, Kangwon National University, Chuncheon 24341, Republic of Korea; gobiobotia@kangwon.ac.kr; 2 Shin Sung Bio Pharm Inc., Saimdang-ro, Gangneung 25451, Republic of Korea; waterroad79@daum.net; 3 Department of Biomedical Technology, Kangwon National University, Chuncheon 24341, Republic of Korea; dclee@kangwon.ac.kr

**Keywords:** PDRN, adenosine A2A receptor, synergistic effect, biochemical agents, physical stimuli, hyaluronic acid, hydrogels, growth factors, enhancements from extracorporeal shockwave therapy (ESWT)

## Abstract

Polydeoxyribonucleotide (PDRN) activates the adenosine A2A receptor (A2AR), triggering anti-inflammatory signaling and providing essential nucleotides for the salvage pathway, thereby helping bypass metabolic bottlenecks and promoting tissue repair. Combining PDRN with biochemical agents and physical stimuli represents a significant shift in medical treatment, moving from monotherapy to an integrated, multi-target regenerative approach. These combinatorial strategies effectively address the limitations of PDRN, such as its rapid degradation and diffusion, by simultaneously meeting the structural, metabolic, and signaling needs of injured tissues. The mechanism of action for PDRN involves a synergistic effect with hyaluronic acid, amplification of growth factors (e.g., Platelet-Rich Plasma (PRP), Epidermal Growth Factor (EGF), Platelet-Derived Growth Factor (PDGF)), and enhancements from extracorporeal shockwave therapy (ESWT) and lasers. This results in a notable acceleration of the repair process for chronic wounds, musculoskeletal disorders, and neurological injuries. As intelligent delivery systems like responsive hydrogels and sustainable L-PDRN production continue to advance, these synergistic protocols are poised to redefine global standards of care in regenerative medicine and esthetic dermatology. Future clinical success will hinge on the standardization of sequence-specific protocols and large-scale validation to ensure long-term safety and efficacy.

## 1. Introduction

The development of regenerative medicine has been significantly shaped by the discovery and clinical use of polydeoxyribonucleotide (PDRN), a standardized mixture of PDRN polymers derived from the sperm DNA of *Oncorhynchus mykiss* (Salmon Trout) or *O. keta* (Chum Salmon) [1]. PDRN has a molecular weight ranging from 50 to 1500 kDa and is produced through a rigorous high-temperature purification process that effectively inactivates proteins and peptides, thereby reducing immunogenic risks to human health [1]. Its therapeutic profile includes tissue repair, anti-ischemic, and anti-inflammatory activities, primarily through the selective activation of the adenosine A2A receptor (A2AR) [1]. In addition to its primary signaling role, PDRN serves as a crucial substrate for the purine and pyrimidine salvage pathways, supplying essential building blocks for DNA synthesis in metabolically stressed or hypoxic tissues [2]. While PDRN’s intrinsic properties are significant, recent clinical and preclinical research indicates that its synergistic combination with biochemical agents, hyaluronic acid, growth factors, and physical stimuli—such as lasers, microneedles, and electrical currents—greatly enhances regenerative outcomes.

## 2. The Pharmacological Foundation of PDRN Synergy

The therapeutic effects of PDRN stem from its dual role as a receptor-targeting pharmacological agent and a metabolic substrate for treating chronic pain and depression [2]. Acting as a prodrug, PDRN is enzymatically degraded by extracellular ecto-nucleotidases and nucleases into biologically active nucleosides, predominantly adenosine [3]. These nucleosides selectively bind to the A2AR on the cell membrane [2], initiating a G-protein-coupled signaling cascade that activates adenylate cyclase and increases intracellular cyclic adenosine monophosphate (cAMP) levels [2]. The rise in cAMP promotes the phosphorylation of the cAMP response element-binding protein (p-CREB), a key transcriptional regulator that drives the expression of anti-inflammatory mediators and pro-angiogenic factors [2,4]. This pathway activation suppresses the release of pro-inflammatory cytokines, including tumor necrosis factor-α (TNF-α), interleukin-6 (IL-6), and high-mobility group box 1 (HMGB-1), while enhancing the production of interleukin-10 (IL-10) and vascular endothelial growth factor (VEGF) [1,2,5,6].

In addition to its receptor-mediated effects, PDRN acts as a nucleotide source for the salvage pathway, an energy-efficient mechanism for nucleic acid synthesis, especially when de novo nucleotide synthesis is impaired [1]. In injured or ischemic tissues, de novo nucleotide synthesis often suffers due to the high metabolic demands involved in purine and pyrimidine ring formation [2]. By providing pre-formed nucleotide fragments, PDRN facilitates rapid DNA repair and encourages cellular progression into the proliferative phase [2]. This metabolic support is particularly crucial for fibroblasts, keratinocytes, osteoblasts, and endothelial cells during tissue regeneration and remodeling [2]. This metabolic contribution is particularly critical for fibroblasts, keratinocytes, osteoblasts, and endothelial cells during tissue regeneration and remodeling [2].

The biological activity of PDRN is also influenced by its conformational characteristics [7]. In PDRN preparations, single-stranded DNA (ssDNA) random coils demonstrate significantly greater antioxidant, anti-inflammatory, and anti-aging effects compared to double-stranded helical structures [7]. Notably, ssDNA fragments show 3.9-fold greater inhibition of elastase and 2.2-fold greater inhibition of collagenase than conventional PDRN, highlighting the importance of polymer structural distribution in achieving synergistic efficacy, particularly in dermatological applications [7].

PDRN is a powerful A2AR agonist, but its effectiveness as a standalone treatment may be limited by local tissue conditions and rapid degradation in the extracellular space. To address this, incorporating PDRN into delivery systems such as hydrogels or nanoparticles can protect it from nuclease-mediated degradation and facilitate sustained release at target cells [8]. Additionally, when administered alone, PDRN may quickly diffuse away from the treatment area. Techniques such as microneedling or iontophoresis can enhance tissue penetration by overcoming barriers like the stratum corneum and fibrotic tissue, thereby improving access to deeper tissue compartments that are harder to detect [9,10].

Tissue regeneration is complex and typically involves multiple signaling pathways rather than relying on a single pathway. While PDRN mainly promotes anti-inflammatory and pro-angiogenic effects through the activation of the salvage pathway, treating complex wounds and degenerative conditions often necessitates a combination of therapeutic strategies. Co-administering PDRN with growth factors, such as VEGF or transforming growth factor-β (TGF-β), or with hyaluronic acid (HA), creates a “multi-hit” therapeutic effect by providing metabolic substrates, structural scaffolding, and directional biochemical cues simultaneously [10,11]. Concurrently, physical modalities such as extracorporeal shockwave therapy (ESWT) and low-level laser therapy (LLLT) improve membrane permeability and local microcirculation, thereby enhancing cellular responsiveness to PDRN-mediated signaling and potentially boosting overall regenerative outcomes [12,13].

## 3. Synergistic Combinations with Biochemical and Physical Agents

The integration of PDRN with other bioactive substances or physical stresses aims to establish a multi-target therapeutic environment that addresses both the structural and cellular needs of tissue repair.

### 3.1. Viscoregenerative Synergy: HA and PDRN

The combination of PDRN and HA is widely recognized as a highly effective synergistic approach in regenerative medicine [10,11]. HA primarily provides mechanical support through its viscoelastic, lubricating, and hydrating properties, while PDRN delivers essential biological signals for tissue repair and regeneration [10,11].

In knee osteoarthritis, intra-articular co-administration of PDRN and HA has shown superior clinical outcomes compared with HA alone, particularly in patients with chronic osteoarthritis, who are at increased risk of the condition. Those receiving combination therapy experience greater reductions in pain, as indicated by the visual analog scale, and more significant improvements in functional indices, such as the Western Ontario and McMaster Universities Osteoarthritis Index and Knee Society Score, over six months [14,15].

This enhanced efficacy is attributed to their complementary mechanisms: HA restores the joint’s mechanical environment by improving lubrication and shock absorption, while PDRN promotes the synthesis of the extracellular matrix (ECM) in human chondrocytes, including type II collagen and aggrecan [2], and suppresses matrix metalloproteinase (MMP) activity responsible for cartilage degradation [2]. In addition, PDRN exerts anti-inflammatory effects through the A2AR-mediated upregulation of IL-10 and reduction in pro-inflammatory cytokines such as TNF-α, thereby addressing both structural and inflammatory components of joint degeneration [14,15].

The synergistic principle extends beyond osteoarthritis to applications in esthetics and skin regeneration. In facial rejuvenation protocols that combine HA, PDRN, and calcium hydroxyapatite, HA delivers immediate hydration and volume restoration, while PDRN promotes long-term collagen synthesis and tissue remodeling [10,11]. For wound healing and scar management, HA acts as a temporary scaffold that facilitates cell migration and stabilizes the extracellular environment. When used alongside PDRN, it enhances barrier repair and accelerates tissue reconstruction [16,17].

Additionally, incorporating PDRN into HA-based hydrogels improves drug delivery efficiency and minimizes the risk of side effects. Since PDRN has a relatively short half-life, HA matrices enable sustained release and prolonged regenerative stimulation. This is particularly beneficial for chronic wounds, diabetic ulcers, and cartilage defect repair [10,18].

Table 1 summarizes the mechanisms and synergistic effects resulting from the combination of HA and PDRN.

### 3.2. PLGA and PDRN Synergy

Poly-lactic-co-glycolic acid (PLGA) is a synthetic polymer known for its excellent biodegradability and biocompatibility, making it a popular choice as a scaffold material in tissue engineering. However, PLGA has inherent limitations that complicate the production of polymeric acid. During degradation, it generates acidic byproducts, such as lactic acid and glycolic acid, which can lower the local pH and trigger inflammatory responses. Additionally, PLGA has relatively low mechanical strength and a high nitrogen content [26,27,28,29].

The incorporation of PDRN helps to overcome these limitations through both biological and physicochemical mechanisms, creating a more effective regenerative platform for developing new therapies for various diseases. However, because PDRN is water-soluble and has a short in vivo half-life of approximately 3 h, it is challenging to maintain therapeutic concentrations at the target site without administering high doses.

Encapsulating PDRN within a PLGA carrier enables sustained release over several weeks, thereby promoting prolonged regenerative stimulation [4,29]. Furthermore, PDRN mitigates PLGA-induced acid-mediated inflammation by activating A2ARs, which leads to the downregulation of pro-inflammatory cytokines [26,27,29]. Meanwhile, PLGA provides essential mechanical support and creates a three-dimensional microenvironment that facilitates cell adhesion and proliferation in tissue engineering applications [4,10].

In this composite scaffold, MH neutralizes acidic byproducts to reduce local inflammation and enhances mechanical strength, while the bECM and demineralized bone matrix mimic the native bone microenvironment, offering superior osteoconductive and osteoinductive properties [26,27]. PDRN, an adenosine A2AR agonist, enhances regeneration by increasing VEGF expression, which promotes neovascularization, and by inducing macrophage polarization toward the M2 phenotype, thereby modulating the immune response [26,29].

This synergistic interaction reduces excessive osteoclast activity while boosting osteogenic gene expression and mineralization in mesenchymal stem cells within the hippocampus [26,29]. This synergistic interaction reduces excessive osteoclast activity while boosting osteogenic gene expression and mineralization in mesenchymal stem cells within the hippocampus [26,27]. As a result, this multifunctional hybrid scaffold demonstrates effective regeneration and functional recovery in complex bone defect models, such as calvarian defects and spinal fusion, underscoring its potential as a promising therapeutic strategy [26,29].

Table 2 summarizes the mechanisms and synergistic effects of the PLGA-PDRN combination.

### 3.3. Synergy of PDRN and Biochemical Agents

The healing of chronic diabetic wounds is a complex process often complicated by persistent inflammation, bacterial infection, and insufficient angiogenesis [18]. To address these challenges, a multifunctional composite nanoparticle was developed that integrates PDRN and stem cell-derived exosomes with silk fibroin and ε-poly-L-lysine (EPL). This nanoparticle produces a single polymerase of PDRN and exhibits potent, broad-spectrum antibacterial activity through EPL, effectively suppressing infections at the wound site [18]. Additionally, the synergistic effects of PDRN and exosomes promote angiogenesis by inducing macrophages to express the anti-inflammatory M2 phenotype and supporting the proliferation of vascular endothelial cells. In experimental animal models, this complex significantly accelerated the closure of diabetic wounds by enhancing granulation tissue formation and collagen deposition. This integrated biological-function nanoplatform offers a promising clinical strategy for treating chronic diabetic wounds [18].

Recent studies have investigated combining PDRN, known for its excellent tissue-regenerative properties, with natural polymer-based dressings that exhibit high biocompatibility to enhance wound healing [25,31]. Bacterial cellulose, characterized by an aligned nanofiber structure, provides topographical signals that encourage cell migration and the formation of new cellular structures. When paired with PDRN, it promotes cell proliferation and angiogenesis, significantly accelerating wound healing and reducing the risk of complications such as multiple sclerosis [31]. The incorporation of MXene into silk fibroin-based hydrogels enhances mechanical strength and antimicrobial activity, enabling precise control of PDRN release via near-infrared (NIR) light. This maximizes infection prevention and enhances regenerative efficiency while minimizing infection risk [25]. These composite dressing systems facilitate the functional recovery of skin tissue by reducing inflammatory responses and promoting re-epithelialization and collagen deposition through the synergistic effects of their physical structure and biologically active components [25,31]. Consequently, gene transcription and histological analyses have shown that these smart dressings activate actin-related biological processes, leading to rapid wound closure and cell death in patients with multiple sclerosis [25,31].

When PDRN and demineralized dentin matrix (DDM) were co-implanted into the soft tissue of nude mice, they successfully induced the differentiation of osteoblasts and fibroblasts, leading to new bone formation [32]. The most significant new bone formation occurred at two weeks post-implantation, attributed to the synergistic effects of PDRN’s ability to stimulate cell proliferation and the supportive scaffold properties of DDM. PDRN promotes tissue regeneration by activating the A2AR, while DDM provides an optimal environment for bone regeneration, serving as a growth-inductive material for bone tissue repair. Additionally, DDM is an effective biomaterial for reconstructing bone defects in the oral and maxillofacial regions. Thus, the combination of PDRN and DDM shows great promise as a biomaterial for addressing bone defects in these areas [32].

Ultraviolet B exposure induces oxidative stress in the skin, which is a primary contributor to increased melanin production and decreased skin elasticity [33]. A mixture of PDRN, vitamin C, and niacinamide has been shown to be effective for anti-aging and reducing pigmentation [9]. This mixture activates the NRF2/HO-1 pathway and inhibits the expression of MMPs, preventing the degradation of collagen and elastin fibers [33]. Additionally, regulating the expression of mitochondrial nicotinamide nucleotide transhydrogenase (NNT) helps reduce oxidative stress and maintain intracellular reducing states (GSH, NADPH) [9]. As a result, the treatment significantly decreases melanin synthesis and improves skin pigmentation by inhibiting microphthalmia-associated transcription factor (MITF) and tyrosinase activity [9,33]. Notably, using a microneedle therapy system enhances the penetration of these active ingredients into the skin, maximizing their effectiveness [9,33].

The effects of PDRN and atelocollagen, both as monotherapy and in combination, on tissue regeneration and biomechanical recovery in Achilles tendon injuries were investigated [34]. After a four-week observation period in an experimental rat model, the group receiving the combined PDRN and atelocollagen treatment exhibited a significantly improved energy absorption capacity in the damaged tendon compared to the control group. This finding was consistent with previous studies. Histological examination indicated that the combined therapy reduced neutrophil infiltration and promoted collagen fiber production and fibroblast proliferation, facilitating the resolution of inflammation and tissue regeneration. Immunohistochemical analysis showed that the combination therapy accelerated tendon regeneration by increasing the expression of key healing growth factors, including Collagen I, TGF-β1, VEGF, and fibroblast growth factor (FGF). In summary, the combined use of PDRN and atelocollagen exhibits superior synergistic effects compared to monotherapy, suggesting its potential as an effective clinical alternative for treating tendon injuries [34].

A novel treatment that combines air-assisted botulinum toxin injections with PDRN has recently been proposed to enhance scar-reconstruction effects in acne scar therapy [35]. This approach creates therapeutic synergy by integrating the skin remodeling and collagen-synthesis effects of botulinum toxin with the tissue-regeneration and angiogenesis mechanisms of PDRN. Clinical trial results showed that the combined therapy group experienced a significant 12.0-point reduction in POSAS scores, indicating a greater improvement than monotherapy with either botulinum toxin (9.0 points) or PDRN (6.1 points). Additionally, the patient satisfaction rate was highest in the combined therapy group, and the treatment was administered safely, with no serious adverse effects beyond mild edema or erythema [35]. In conclusion, these findings suggest that this combined therapy, which leverages both mechanical and biological synergies, has the potential to be a more effective alternative for managing acne scarring and treating acne and other related conditions [35].

Melatonin serves as a regulatory molecule with strong antioxidant and anti-inflammatory properties, while PDRN is a bioactive compound that promotes tissue regeneration and angiogenesis [36]. When combined, these two components create a synergistic effect, enhancing their pharmacological benefits to establish a more effective antioxidant defense system and a conducive environment for tissue repair [36]. Notably, biomimetic scaffolds incorporating melatonin and PDRN have emerged as a promising therapeutic approach for the functional recovery of ovarian tissue in models of early ovarian failure induced by anticancer chemotherapy [37]. Within these scaffolds, PDRN promotes angiogenesis, ensuring adequate oxygen and nutrient supply, while melatonin mitigates oxidative stress and inflammation, preventing cell death and fostering follicle formation [37]. The effectiveness of this combined system has been demonstrated in experiments showing reduced ovarian fibrosis, normalized hormone secretion, significant restoration of fertility, and increased offspring numbers [37]. In conclusion, the interaction between melatonin and PDRN holds the potential to provide innovative therapeutic options across various medical fields, including regenerative medicine and dermatology [36,37].

Acute Respiratory Distress Syndrome (ARDS) is characterized by severe hypoxemia and pulmonary edema, with the subsequent development of pulmonary fibrosis being a critical factor that increases patient mortality [38]. PDRN exhibits potent anti-inflammatory effects by reducing the production of inflammatory cytokines, while pirfenidone inhibits the progression of pulmonary fibrosis by modulating collagen synthesis and growth factor signaling. Compared with monotherapy, the combination of PDRN and pirfenidone has shown greater effectiveness in suppressing fibrosis markers, such as connective tissue growth factor and hydroxyproline, and inflammatory mediators such as TNF-α and IL-6 [38]. The efficacy of this combination therapy is evident in its ability to significantly alleviate ARDS symptoms through a dual action: rapidly inducing an anti-inflammatory response and inhibiting the fibrotic process. In summary, the combined use of PDRN and pirfenidone offers substantial promise for overcoming the limitations of current treatments and presents new therapeutic guidelines and strategies for patients with ARDS [38].

Glucosamine is a commonly used anti-inflammatory agent for treating knee osteoarthritis. When combined with PDRN, it is utilized in anti-aging and bio-stimulation therapies [23]. Co-administering both substances to dermal fibroblasts resulted in a significant suppression of MMP13 expression, which is crucial for cartilage degradation, as well as the IGF-I gene, which is involved in tissue regeneration, after just 24 h of treatment with a single dose. This outcome contrasts with the activation of MMP13 or the modest inhibition of IGF-I observed when glucosamine or PDRN is used alone, highlighting the unique synergistic effect of the combination therapy, which enhances MMP13 efficacy. Furthermore, the PDRN and glucosamine combination was found to optimize the extracellular matrix by modulating the expression of the neutrophil elastase ELANE and desmoplakin DSP genes. These findings suggest that the glucosamine and PDRN combination could be a promising new therapeutic option for orthopedic cartilage treatment [23].

### 3.4. Growth Factor Amplification: PRP, EGF, and PDGF

The combination of PDRN and various growth factors creates a robust regenerative response by merging structural support with cellular instructions to effectively combat disease [5,6,39]. PDRN acts as the “architect,” providing essential blueprints and raw materials (nucleotides) for tissue repair, while growth factors function as “coaches” that guide cellular growth and renewal [5,6,39]. By serving as a catalyst, PDRN enhances the effects of both externally administered and naturally secreted growth factors, significantly improving the body’s regenerative capacity.

In esthetic and skin-repair applications, PDRN optimizes the effectiveness of standard treatments and bolsters their underlying mechanisms. For instance, PRP delivers a concentration of cytokines (VEGF, PDGF, and TGF-beta) that promote angiogenesis and fibroblast activity [40,41]. When combined with PDRN, the biological scaffold of the plasma is enhanced, as PDRN supplies the nucleotides necessary for the rapid proliferation of cells stimulated by PRP to generate the desired proteins [40,41]. For patients with Female Pattern Hair Loss (FPHL), the combination of PRP and PDRN yields better results in hair density and thickness than PDRN alone [41]. This improvement is attributed to PRP’s ability to upregulate growth factors such as PDGF, FGF9, and WNT, which work in conjunction with PDRN to stimulate follicle cells and promote blood vessel formation [41]. Furthermore, incorporating PDRN into the growth factor serum accelerates post-procedure healing [5,6,39]. Specifically, the combination of PDGF and PDRN in microneedling protocols “supercharges” regeneration, improving skin elasticity and radiance while minimizing downtime and the risk of skin damage [5,6,39].

Beyond surface treatments, PDRN facilitates deep tissue and skeletal recovery. It binds to bone morphogenetic protein (e.g., BMP2), synergistically enhancing the expression of genes responsible for bone differentiation and angiogenesis [10,27]. This crucial interaction enables effective bone regeneration even at low BMP2 concentrations, thereby minimizing the risk of side effects, such as ectopic bone formation, associated with high-dose BMP2 [10,27]. In the treatment of chronic ulcers, epidermal growth factor (EGF) promotes keratinocyte migration, while PDRN supports underlying dermal repair through A2AR activation and the salvage pathway [2]. Additionally, PDRN degradation products directly enhance the activity of EGF and PDGF, resulting in faster wound closure and superior recovery of tensile strength compared to growth factors alone [33,42]. By stimulating the A2AR, PDRN induces the release of VEGF from cells, which is vital for treating atrophied muscles or ruptured tendons, where limited blood supply typically hinders recovery [33,36,43].

In conclusion, the mechanisms and synergistic effects of combining biochemical agents or growth factors with PDRN are summarized in Table 3.

## 4. Synergy with Physical and Electrical Stimuli

The combination of PDRN with physical modalities examines the intersection of mechanotransduction, thermal energy, and bioelectrical signaling to optimize tissue repair [12,42]. These strategies typically aim to enhance PDRN delivery or leverage the physiological responses activated by physical stress.

### 4.1. Mechanical Delivery and Micro-Injury: Microneedling and PDRN

Microneedling improves PDRN absorption by creating microchannels in the skin that bypass the natural skin barrier. This process initiates a healing response, enhancing regenerative effects. The microchannels enable efficient delivery of large PDRN molecules into the dermis, thereby improving drug delivery efficiency [9,10,21,33,47]. Research indicates that microneedling with PDRN effectively reduces acne scars, fine lines, and hyperpigmentation [21,33,47]. The micro-injuries from microneedling stimulate collagen and elastin production, and, when combined with PDRN, the treatment helps skin cells regenerate more effectively [9,10,21,33,47]. The benefits of this treatment are further enhanced by the addition of radiofrequency energy, which stimulates collagen production through controlled heat. Meanwhile, PDRN supplies the necessary materials for cellular repair and reduces inflammation. This combination effectively breaks down scar tissue and promotes the regeneration of skin fibers, particularly for atrophic scars [2,21,33]. In treating acne scars, air-assisted injection technology using microneedles combines physical exfoliation with PDRN’s regenerative effects to promote collagen synthesis [2]. Additionally, using a microneedle platform alongside microcurrent therapy improves the immune environment at chronic wound sites and accelerates re-epithelialization [24], while delivering PDRN with whitening ingredients is more effective at inhibiting melanin production than topical applications [9].

### 4.2. Thermal Energy and Micro-Treatment Zones (MTZ) Remodeling: Laser Therapy and PDRN

Combining PDRN with fractional CO2 lasers and light-based therapies, such as Broad Band Light and Halo Pro, effectively addresses the limitations of thermal resurfacing [48]. While fractional lasers create microthermal zones that stimulate collagen remodeling, they can also disrupt the skin’s barrier [48]. While fractional lasers create microthermal zones that stimulate collagen remodeling, they can also disrupt the skin’s barrier [48]. Laser treatment creates thermal channels that enhance PDRN absorption by preparing the skin, while PDRN in turn accelerates the healing of these micro-wounds. Consequently, the immediate application of PDRN after laser treatment has been shown to expedite epidermal regeneration and granulation tissue formation, significantly reducing recovery time [13,48,49]. Research shows that PDRN injection following CO_2_ fractional laser therapy increases VEGF expression and vascularization, promoting robust tissue remodeling in atrophic scars [48].

LLLT and PDRN also demonstrate synergistic effects in facial nerve injury models, enhancing blood flow and restoring nerve conduction, which fosters nerve regeneration [13]. Furthermore, NIR-responsive MXene-based hydrogel systems release PDRN upon laser stimulation, enabling precise delivery of high-concentration medication to wound sites [24,25], while PDRN applied to MTZ suppresses inflammation and aids rapid tissue reconstruction [10,49].

### 4.3. Mechanotransduction and Acoustic Waves: ESWT and PDRN

ESWT generates acoustic waves that activate mechanotransduction pathways, stimulating metabolic activity and tissue remodeling [12]. Research shows that the combination of PDRN and ESWT is more effective than either treatment alone for muscle atrophy, particularly when ESWT follows PDRN injection. This sequence leads to greater improvements in muscle regeneration, fiber cross-sectional area, and electrophysiological recovery. The synergistic effect is believed to arise from shock wave-induced cavitation and shear stress, which temporarily increase cell membrane permeability [12], facilitating enhanced PDRN penetration and boosting growth factor activation and gene expression. Histological analyses further reveal that this combined treatment significantly upregulates the expression of cell nuclear antigen and VEGF, thereby promoting neovascularization and muscle fiber growth in cast-immobilized rabbit models [12]. Similarly, in models of cast-immobilization-induced muscle atrophy, the combination of ESWT and PDRN injection significantly increases cross-sectional area and improves functional recovery compared to monotherapy [12,50]. Mechanistically, shock wave-induced microbubble destruction (cavitation) temporarily increases tissue membrane permeability, thereby enabling deeper PDRN delivery [12,50]. This combined approach also synergistically elevates VEGF and platelet endothelial cell adhesion molecule-1 (PECAM-1) expression, promoting regeneration in poorly vascularized tendon and muscle tissues [12,50]. Additionally, applying ESWT after PDRN injection yields more pronounced regenerative effects across key parameters than when the treatments are administered in the reverse order [12].

### 4.4. Bioelectrical Signaling: Microcurrent Therapy and Nerve Regeneration

Microcurrent electrical neuromuscular stimulation and continuous microcurrent electrical nerve stimulation transmit bioelectrical signals that mimic the body’s natural physiological currents [51,52]. By promoting satellite cell activation during regeneration, microcurrent electrical neuromuscular stimulation aids in repairing injured skeletal muscle and regrowing atrophied fibers [53,54]. The therapeutic interaction between these modalities and PDRN is based on complementary mechanisms: PDRN provides biochemical substrates and activates repair pathways through A2AR, while microcurrents modulate the cellular microenvironment and enhance ion transport [2].

In models of peripheral nerve defects, continuous microcurrent electrical nerve stimulation enhances Schwann cell activity and increases neurotrophic factor secretion, thereby promoting axonal regeneration and reducing denervation-induced muscle atrophy [51]. Among waveform types, sine-wave microcurrents have shown superior efficacy due to their greater conductivity in deeper muscle tissue and lower discomfort compared with other waveforms [55]. Together, these findings support a combined PDRN-microcurrent protocol as a dual-modality strategy for neuromuscular rehabilitation, merging metabolic support with bioelectrical stimulation to enhance functional recovery [51,52].

Microcurrent stimulation also enhances intracellular ATP production, creating a high-energy environment that facilitates PDRN-mediated tissue repair and protein synthesis [20,56]. In a chronic rotator cuff tear model, the simultaneous injection of PDRN and the application of microcurrents reduced the tear size and accelerated type I collagen regeneration [20,56]. Similarly, in cases of muscle atrophy due to nerve paralysis, the combined treatment improved compound muscle action potential and enhanced motor functional recovery [20]. Additionally, when used alongside stem cell-derived exosomes, this approach boosts angiogenic effects, indicating its potential as a multifaceted therapy for neurological and musculoskeletal disorders [10,20].

The mechanisms and synergistic effects of combining physical and electrical stimuli with PDRN are summarized in Table 4.

## 5. Therapeutic Applications and Clinical Evidence

The clinical utility of PDRN-based synergistic therapies spans a wide range of medical specialties, from chronic wound care to neuroprotection and esthetic dermatology [2].

### 5.1. Chronic Wound Healing and Diabetic Ulcers

PDRN is most clinically established for managing chronic wounds, especially diabetic foot ulcers [1], and has demonstrated improved healing rates in chronic diabetic foot ulcers, pressure ulcers, and venous leg ulcers [2]. A large multicenter trial reported a 37.3% complete wound closure rate for Wagner grade 1–2 ulcers treated with PDRN (perilesional and intramuscular injections), compared to 18.9% in the control group. The median percentage of ulcer re-epithelialization was also significantly higher in the PDRN group (82.2% vs. 49.3%) [2].

The synergistic benefits of PDRN in wound care are often observed when it is used alongside standard debridement and advanced dressings containing collagen or HA [2]. PDRN facilitates a quicker transition from the inflammatory to the proliferative phase by modulating cytokine profiles and enhancing the nucleotide pool for DNA repair in the hypoxic wound bed, which is crucial for healthy granulation tissue formation and subsequent epithelialization [2].

PDRN functions as an A2AR agonist, inducing VEGF expression and promoting new blood vessel formation to normalize blood supply at the wound site [10,18]. In diabetic mouse models, PDRN injections supported fibroblast proliferation and significantly accelerated wound closure rates [10,18]. Recent studies are actively combining PDRN with MXene-based hydrogels and directional nanofiber scaffolds for sustained drug release and guided cell migration at chronic ulcer sites [24,25].

### 5.2. Musculoskeletal and Tendon Regeneration

PDRN has emerged as a promising treatment in orthopedics for tendon disorders, including Achilles tendinopathy, rotator cuff disease, and plantar fasciitis [58]. It enhances tendon repair by inhibiting inflammation and cell apoptosis while promoting collagen production [59]. When used alongside physical therapies such as ESWT or guided exercise, PDRN provides a biological stimulus to compensate for the poor vascularization of tendon tissue [12].

Research on atrophied muscles indicates that PDRN and ESWT sequences can significantly improve muscle volume and function after immobilization [12]. Electrophysiological recovery, measured through compound muscle action potential, is notably better in combination treatment groups, reflecting enhanced nerve-to-muscle signaling [12,20]. This suggests that PDRN and its synergistic partners can effectively address both structural and functional aspects of musculoskeletal injuries.

In treating chronic rotator cuff tears, PDRN accelerates functional recovery by promoting type I collagen synthesis and enhancing tensile strength at the tear site [20,60]. In muscle atrophy models resulting from cast immobilization, PDRN injections significantly increased the cross-sectional area of muscle fibers and inhibited atrophy [12,20]. Additionally, the co-administration of hyaluronic acid and PDRN in patients with osteoarthritis demonstrates synergistic effects on pain relief and joint function. During spinal fusion surgery, PDRN also enhances bone fusion rates by working in conjunction with bone morphogenetic protein (e.g., BMP2) [14,27,29].

### 5.3. Neuroprotection and Spinal Cord Injury

PDRN’s neuroprotective role is an active area of research, with significant findings in models of spinal cord injury and cerebral ischemia [4]. Systemic administration of PDRN following spinal cord injury has been shown to reduce tissue damage, demyelination, and motor deficits [61]. This protective effect is mediated by A2AR activation, which stimulates the Wnt/β-catenin pathway and reduces pro-inflammatory cytokines such as TNF-α and IL-1β [61].

In models of cerebral ischemia, PDRN has been shown to improve memory impairment by reducing hippocampal inflammation and inactivating MAPK signaling [4]. The underlying mechanism involves the activation of the cAMP/p-CREB pathway, which supports synaptic formation and protects neurons from excitotoxicity. This suggests that PDRN could be an effective component of multimodal neuroregenerative strategies to prevent secondary damage following acute neurological injuries [4].

PDRN facilitates the restoration of nervous system function by inhibiting neural tissue death and regulating inflammation. In facial nerve injury models, PDRN promotes peripheral nerve regeneration by enhancing blood flow around the nerves and improving nerve conduction velocity [13]. At spinal fusion sites, PDRN-loaded scaffolds encourage M2 macrophage polarization, thereby suppressing excessive inflammation and supporting successful bone tissue union [29]. Additionally, PDRN aids regeneration at both central and peripheral nervous system injury sites by protecting cells from oxidative stress and inhibiting apoptosis-related pathways [10,13].

### 5.4. Esthetic Dermatology and Skin Longevity

In esthetic dermatology, PDRN is increasingly combined with microneedling, lasers, and chemical peels to promote “skin longevity” [21,24,33,47]. This approach focuses on genuine tissue regeneration rather than merely masking signs of aging [21,24,33,47]. By activating fibroblasts and enhancing collagen and elastin synthesis, PDRN results in firmer, smoother skin [62]. When administered via microneedling, it yields more noticeable and longer-lasting effects than microneedling alone [21,24,33]. Additionally, synergies between niacinamide and peptides have been explored to tackle hyperpigmentation and restore skin barrier function [9].

Overall, PDRN plays a vital role in skin regeneration and anti-aging by increasing collagen density and strengthening the skin barrier. It stimulates dermal fibroblasts to enhance collagen and elastin production, improving elasticity and reducing fine wrinkles [10,16]. When used in conjunction with botulinum toxin or laser treatments for acne or hypertrophic scars, PDRN helps reduce fibrosis and gently remodel skin texture [35,49]. Novel variants, such as Lactobacillus-derived PDRN (L-PDRN), show promise for delaying skin aging and promoting long-term skin health due to their improved absorption and antioxidant properties [9,10,63].

The therapeutic applications and clinical evidence of PDRN are summarized in Table 5.

## 6. Insights and Second-Order Effects of PDRN Synergy

The analysis of PDRN synergy reveals trends that indicate a shift toward precision in metabolic and signaling treatments in medicine [2].

### 6.1. The Metabolic Efficiency Hypothesis

One of the most profound insights into PDRN synergy is the role of the salvage pathway as a “metabolic bypass” [2]. In many chronic conditions, such as diabetic ulcers and aging skin, cells experience metabolic exhaustion [2]. By supplying pre-formed nucleotides, PDRN alleviates the energetic bottleneck of de novo synthesis [2]. When used alongside physical stimuli such as microneedling or lasers, which increase cellular repair demand, PDRN ensures that cells have the necessary resources to meet that demand without further depleting their ATP stores [64]. This metabolic efficiency is a key factor driving the accelerated healing observed in combination therapies [2].

### 6.2. Inflammatory Curation and Resolution

PDRN does not simply suppress inflammation; it actively guides the inflammatory response toward resolution [64]. In contrast to corticosteroids, which broadly inhibit immune activity and may hinder long-term healing, PDRN activates A2AR, shifting the cytokine balance from pro-inflammatory to anti-inflammatory [2]. This shift facilitates a “clean” healing process, allowing tissue repair to occur without the detrimental effects of chronic inflammation [64]. This effect is further enhanced when combined with physical modalities that enable deeper delivery or provide mechanical signals that promote the anti-inflammatory phenotype [12].

### 6.3. Sequence-Dependent Synergy

The discovery that the order of therapy—such as administering ESWT after PDRN injection—affects outcomes indicates that their interaction is not merely additive but dynamically integrated [12]. The physical stimulus seems to prepare the tissue or improve the pharmacokinetics of the biochemical agent [12]. This underscores the need for standardized, sequence-specific clinical protocols to optimize the “bio-stimulation” effect [12].

## 7. Future Outlook in PDRN-Based Regenerative Medicine

Combination therapy that integrates PDRN with various treatment modalities is creating strong synergies in regenerative medicine. Its potential is expected to grow further by incorporating intelligent drug delivery systems, combining with biologically active substances, and responding to physical stimuli.

### 7.1. The Evolution of Next-Generation Drug Delivery Systems and Bio-Platforms

■ Smart hydrogels and microneedles: To overcome PDRN’s short half-life (~3 h) and rapid degradation issues, MXene-based hydrogels or soluble microneedle platforms that release drugs only when needed in response to external stimuli, such as NIR light, are emerging as key technologies. These systems will maximize therapeutic efficacy by maintaining high local drug concentrations [24,65].
■ Targeted nanocarriers: Oral delivery technologies utilizing natural nanocarriers, such as tea-derived extracellular vesicles, protect drugs from gastric acid degradation and selectively deliver them to inflammatory sites [66]. This approach represents a new paradigm for treating internal organ diseases, such as colitis.
■ Biomimetic scaffolds: Research that combines directional nanofiber scaffolds (aligned scaffolds) to guide cell migration, demineralized dentine matrix, and PDRN will enhance the precision of tissue regeneration and accelerate it.

### 7.2. Maximizing Synergy with Biologically Active Substances

■ Combination with exosomes and cell-derived materials: The co-administration of stem cell-derived exosomes and PDRN will synergistically enhance immunomodulatory and angiogenic effects, providing a powerful therapeutic approach for challenging conditions such as chronic wounds, renal regeneration, and spinal fusion.
■ Research into novel composite formulations: The combination of melatonin, known for its strong antioxidant and anti-inflammatory properties, with PDRN is attracting attention as a novel therapeutic strategy for pain management and anti-aging. The concurrent use of atelocollagen is expected to prolong the drug’s residence time, thereby enhancing the regenerative effects on tendons and ligaments.

### 7.3. Diffusion of Combined Therapies with Physical Energy

■ Energy-based adjunctive therapies: Physical stimuli, such as ESWT, microcurrent and LLLT, synergistically enhance tissue permeability and induce VEGF expression. This “energy-drug fusion model” is expected to become the standard for treating muscle atrophy and promoting nerve regeneration.

### 7.4. Challenges for Source Material Innovation and Clinical Commercialization

■ Securing Sustainable Materials: Beyond traditional salmon-derived methods, L-PDRN production using microorganisms like Lactobacillus is poised to become a future technology that offers both high bioavailability and environmental sustainability.
■ Standardization and Large-Scale Clinical Validation: Standardization of extraction processes and stringent quality control are essential for the widespread use of PDRN-based combination therapies in clinical practice. Additionally, conducting large-scale, multicenter randomized controlled trials to demonstrate long-term safety and efficacy across various disease models will be a pivotal step in determining future commercialization.
■ Regulatory compliance: Ongoing multidisciplinary research is essential to meet the dual regulatory requirements for convergent products that combine drugs and medical devices, as well as to establish optimal dosage settings.

Therefore, the future of PDRN combination therapy is expected to move beyond basic drug administration toward a cohesive regenerative system. In this system, the ‘carrier-drug-physical stimulus’ will be precisely integrated, leading to significantly improved treatment outcomes in clinical practice.

## 8. Conclusions

**Figure 1:**
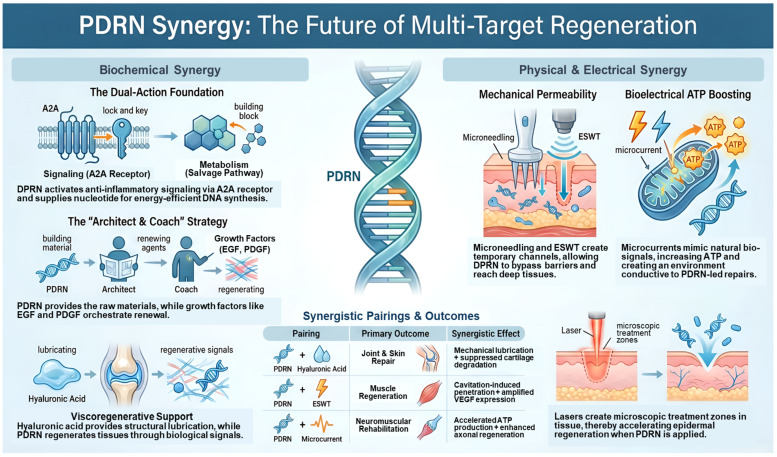
Summary of enhancing PDRN efficacy through biochemical and physical modalities.

The integration of PDRN with biochemical agents and physical stimuli marks a significant shift in regenerative medicine, evolving from simple monotherapy to a sophisticated, multi-target therapeutic system. The clinical effectiveness of PDRN is based on its unique dual-action profile: it selectively activates A2AR to initiate anti-inflammatory and pro-angiogenic signaling cascades, while also serving as a crucial metabolic substrate for the salvage pathway. This role provides essential nucleotides for DNA synthesis in tissues experiencing metabolic or hypoxic stress. PDRN’s effectiveness increases when combined with structural carriers or physical energy, which help address its short physiological half-life and rapid diffusion from target sites. The concept of viscoregenerative synergy, particularly evident in the combination of PDRN and hyaluronic acid, illustrates the synergistic effect of mechanical lubrication and biological signals, aimed at restoring joint function and accelerating wound healing. Additionally, PDRN acts as a vital catalyst for growth factors such as PRP, BMP2, and EGF, supplying the metabolic “blueprints” that enable these molecules to enhance cellular proliferation and bone mineralization, even at lower, safer doses (Figure 1).

The “energy-drug fusion model,” which incorporates ESWT, lasers, and microcurrents, goes beyond biochemical pairings to create a high-energy environment that maximizes tissue repair. Physical modalities leverage mechanotransduction and cavitation to temporarily increase cell membrane permeability, enhancing the penetration of PDRN. Microcurrents specifically boost intracellular ATP levels, supporting the protein synthesis and tissue remodeling initiated by PDRN (Figure 1). A deeper exploration of these synergies leads to the “Metabolic Efficiency Hypothesis,” which posits that PDRN circumvents the high costs of de novo nucleotide synthesis. Additionally, the concept of “Inflammatory Curation” proposes that the tissue environment actively shifts toward resolution rather than merely suppressing inflammation. Research indicates that the sequence of therapy, particularly administering ESWT after PDRN injection, is crucial for optimizing these integrated interactions. As the field progresses towards intelligent drug delivery systems like stimulus-responsive hydrogels and sustainable L-PDRN, the emphasis will be on standardizing sequence-specific protocols and conducting large-scale clinical validations. These synergistic strategies aim to address the structural, metabolic, and signaling needs of damaged tissue simultaneously, ultimately redefining global standards of care in regenerative medicine and esthetic dermatology. They provide a comprehensive healing environment that significantly surpasses the efficacy of isolated treatments.

## Figures and Tables

**Table 1:** Summary of synergistic roles of HA with PDRN.

Stimulant	Primary Mechanism of Action	Synergistic Role with PDRN	References
HA/Hyaluronate	Binds to CD44 receptors to promote cell proliferation and tissue healing; retains moisture for skin hydration, restores turgidity, and acts as a lubricating, anti-collision molecule and bacterial filter.	Hyaluronate enhances the effect of PDRN on human cultured fibroblasts, improving skin hydration, firmness, and structural integrity while stimulating collagen synthesis and cellular repair.	[10,19]
HA solution/hydrogel	Facilitates delivery of therapeutic agents (e.g., nanoparticles or stem cells) to promote granulation tissue formation and wound epithelialization.	HA serves as a dispersing medium or scaffold for PDRN and UCB-MSCs; the combination enhances M2 macrophage polarization, angiogenesis, and collagen type 1 fiber regeneration in chronic wounds or tendon tears.	[8,10,18]
Intra-articular injection (Viscosupplementation)	Provides joint lubrication and shock absorbency; reorganizes articular cartilaginous structure through regulation of water molecule coordination and viscoelastic action.	The combination stimulates greater ECM production and synoviocyte growth than single agents; PDRN enhances anti-inflammatory actions by elevating IL-10, influencing TNF-*α* and IL-6, and inhibiting proteoglycan degradation.	[14,15,20,21,22]
Glucosamine (Precursor)	Glucosamine and acetic acid form N-acetylglucosamine, which polymerizes with glucuronic acid to produce endogenous HA.	Mesotherapeutic administration of HA precursors with PDRN improves HA production in the dermal compartment and enhances cellular replication and protein synthesis in dermal fibroblasts.	[23]
Micronutrient mixture and NCTF (Vitamins, amino acids, coenzymes, minerals)	Supports dermal hydration and provides a temporary scaffold for cellular migration, creating a metabolically favorable microenvironment for tissue repair.	The combination of NCTF (containing HA) and PDRN accelerates full-thickness skin wound healing by promoting fibroblast proliferation, collagen synthesis, and angiogenesis superior to using either agent alone.	[17]
Near-infrared (NIR)-responsive Microneedle Platform	Facilitates localized drug delivery via an HA matrix that dissolves, releasing bioactive agents directly into the dermis.	HA matrix encapsulates PDRN and MSC-derived nanovesicles; co-delivery leads to synergistic regulation of immunomodulation, angiogenesis, ECM regeneration, and collagen synthesis.	[24]
Visible light-crosslinkable silk fibroin and gelatin/MXene composite	Provides a moist, protective environment that supports cell migration, proliferation, and ECM remodeling.	The HA matrix integrates with PDRN to enhance therapeutic outcomes; PDRN acts as an adenosine A2AR agonist to promote regeneration while HA provides structural support.	[25]

**Table 2:** Summary of Synergistic Roles of PLGA with PDRN.

Stimulant	Primary Mechanism of Action	Synergistic Role with PDRN	References
Ischemic or hypoxic conditions (angiogenesis)	Advanced PLGA hybrid scaffold delivery system for a PDRN/BMP2 nanocomplex.	Synergistically promotes angiogenesis and bone regeneration by utilizing human fetal MSCs and activating A2AR-mediated pathways.	[24,25]
Bone tissue repair or bone defect environment	PLGA-based scaffold (bioinspired PLGA/MH/ECM) acts as a carrier for PDRN to establish a pro-regenerative environment.	Promotes bone regeneration by synergistically enhancing osteoblast proliferation and differentiation.	[24,25]
Critical-sized bone defect environment (hBMSCs)	MH neutralizes acidic byproducts from PLGA degradation; bone extracellular matrix (bECM) provides osteoconductivity by mimicking the natural bone microenvironment.	The PMEP scaffold combines MH, bECM, and PDRN to enhance hBMSC adhesion, proliferation, and osteogenic differentiation; PDRN acts as an A2AR agonist, upregulating VEGF for angiogenesis and down-regulating inflammatory cytokines while inhibiting osteoclastogenesis.	[26]
TNF-α/interferon-γ (IFN-γ)	PLGA serves as a porous, pneumatic microextrusion composite scaffold that provides biophysical cues and structural support for the delivery of PDRN and primed extracellular vesicles.	The combination of PDRN and tissue-derived extracellular vesicles (TI-EVs) in the PLGA scaffold enhances cellular proliferation, promotes angiogenesis (upregulating FGF2, HGF, and VEGF), reduces fibrosis, and alleviates inflammation through M2 macrophage polarization.	[30]
MH and decellularized kidney ECM	PLGA serves as a biodegradable polymer scaffold carrier; MH is incorporated to neutralize acidic byproducts of PLGA degradation, which typically induce local inflammation.	The scaffold facilitates the steady, sustained release of PDRN over 28 days, which binds to A2ARs to promote functional kidney recovery, including structural restoration of functional glomeruli and normalization of BUN/creatinine levels.	[30]
Partial nephrectomy (surgical defect)	The integrated bioactive PLGA scaffold mimics the kidney tissue microenvironment, supporting morphogenetic processes in renal cells.	Collaborates with PDRN to facilitate infiltration of host renal stem/progenitor cells (Pax2-expressing cells) and enhances the glomerular filtration rate (GFR) to native levels.	[30]
Acidic microenvironment (lactic acid and glycolic acid)	MH or modified MH (mMH) acts as a neutralizing agent to counteract the acidic degradation byproducts of PLGA.	Ensures a stable pH environment for PDRN and growth factors, reducing inflammatory responses and enhancing therapeutic efficacy in angiogenesis and bone regeneration.	[10,27,30]
Supersaturated calcium-phosphate (Ca/P) solution	PLGA serves as the main body/scaffold, incorporating bovine inorganic extracellular matrix (bECM) to facilitate ionic interactions.	Facilitates the intensive deposition and immobilization of a negatively charged PDRN/BMP2 nanocomplex onto the scaffold surface for controlled, sustained release.	[10,27,30]
Ionic interaction/Self-assembled nanocomplex	Negatively charged PDRN acts as a carrier/capping agent for positively charged BMP2, forming a self-assembled nanocomplex (NC) on the PLGA scaffold.	Enables sustained release of BMP2 up to 60 days without initial bursts, providing dual effects on angiogenesis and osteogenesis (increased gene expression of ALP, RUNX2, OPN, OCN, and ON).	[10,27,30]
Human fetal mesenchymal stem cells (hfMSCs)	The Advanced PLGA/mMH/bECM hybrid scaffold (PME) provides an interconnected pore structure that supports cell migration and proliferation.	Supports enhanced hfMSC adhesion and differentiation into osteogenic lineages, achieving outstanding performance in vascularization and bone repair in critical-size defects.	[27]
Span 80 (nano-emulsion dispersion method)	Emulsifier achieves homogeneous dispersion of water-soluble PDRN within the hydrophobic PLGA composite matrix.	Ensures stable release and multi-modulation of immune-inflammatory responses, promoting M2 macrophage polarization and improving spinal fusion outcomes.	[29]

**Table 3:** Summary of synergistic combinations of biochemical agents or growth factors with PDRN.

Biological Stimulant	Primary Mechanism	Synergistic Role with PDRN	References
Silk-fibroin and EPL nanoparticles	Exhibiting effective antibacterial activity, inducing an anti-inflammatory M2 macrophage phenotype, and promoting angiogenesis	Promoting granulation tissue formation, collagen deposition, wound tissue epithelialization, and significantly accelerating skin healing	[18]
Bacterial Cellulose (BC)/Aligned Structures	Aligned nanofibrous structure provides a topographic cue (“contact guidance”) to guide fibroblast migration and collagen orientation.	Combines topographic guidance (BC) with bioactive cues (PDRN) to speed up wound healing, promote cell proliferation, and facilitate angiogenesis during skin regeneration.	[31]
MH	Acts as a pH-neutralizing agent to counteract the acidic environment created by PLGA degradation and reduces acute inflammation.	Improves anti-inflammatory properties and ensures sustained release of PDRN and BMP2 within hybrid scaffolds for vascularized bone tissue regeneration.	[27,29]
DDM	Biocompatible scaffold containing type I collagen and BMP to assist in the rapid regeneration of alveolar bone.	DDM serves as an ideal carrier to prevent PDRN absorption and grant manipulability; combined them induce osteoinduction and differentiate cells into osteoblasts for new bone formation.	[32]
Niacinamide (Nicotinamide)	Modulation of NNT and coenzyme levels (NAD^+^/NADPH).	Reduces mitochondrial oxidative stress and enhances melanogenesis via dual signaling pathways (MC1R/MITF, tyrosinase, TYRP1, and TYRP2 signaling); increases skin elasticity by Modulating Nuclear Factor Erythroid 2-like 2 (NRF2)/heme oxygenase-1 (HO-1)	[9,33]
Vitamin C (Ascorbic Acid)	Scavenging free radicals and promoting collagen formation/maturation.	Provides synergistic antioxidant defense and aids in the regeneration of the damaged dermis and basal membrane.	[9,22,33]
Atelocollagen	Low immunogenicity scaffold promotes fibroblast migration and type I collagen synthesis.	Atelocollagen prolongs PDRN effectiveness by reducing early degradation; together, they increase energy absorption in repaired tendons and promote healing growth factors (TGF-β1, VEGF, FGF).	[34]
Air-assisted Botulinum Neurotoxin	Neuromodulation and controlled microtrauma via mechanical air dissection stimulate collagen synthesis.	Air-assisted microtrauma stimulates repair and enhances PDRN penetration, while Botulinum toxin reduces fibrosis, and PDRN enhances biological regeneration.	[35]
Melatonin	Acts via MT1 and MT2 receptors; free radical scavenging.	Enhances anti-inflammatory response and antioxidant defense; synergistically accelerates tissue repair and improves pain management.	[36,37]
Pirfenidone	An anti-fibrotic agent downregulates growth factor expression and suppresses pro-collagen type I and type II production.	Combination therapy exerts a stronger therapeutic effect on ARDS by promoting rapid anti-inflammatory effects and inhibiting fibrotic processes.	[38]
Glucosamine	Amino-sugar involved in glycosaminoglycan and hyaluronic acid synthesis; acts as an anti-inflammatory agent.	The association of Glucosamine and PDRN strongly inhibits MMP13 and IGF-I gene expression in fibroblasts, suggesting potential application in preventing cartilage collagen degradation.	[23]
Proton Pump Inhibitor (Pantoprazole)	Inhibition of gastric acid secretion and reduction of pro-inflammatory cytokines (IL-6, IL-8, TNF-α).	Potently improves tissue regeneration and inhibits pro-inflammatory cytokine production; combination therapy is more effective than monotherapy for gastric ulcer healing.	[43]
L-Arginine	Precursor is synthesized into nitric oxide (NO) by intracellular eNOS/iNOS.	NO production induces endothelialization and vasodilation, promoting the infiltration of PDRN and EVs into tissues for vascular regeneration.	[44]
L-Carnitine	Mitochondrial beta-oxidation of long-chain fatty acids and regulation of energy flow.	Stabilizes membranes during cellular repair processes and modulates fibroblast reduction in skin aging; enhances tensile strength in impaired wounds.	[22]
Calcium ions	Activation of the coagulation process and platelet influx into the dermis.	Triggers the spontaneous release of various growth factors and induces chemotaxis of resident stem compartments toward rapid repairing differentiation.	[22]
Proteolytic enzymes (Papain)	Debriding chemical agent to remove damaged/necrotic tissue; activation of growth factors.	Accelerates nutrient absorption during preparation and activates physiologically present platelet growth factors in damaged tissues.	[22]
Selenium	Anti-inflammatory and anti-oxidative trace element; reduces ROS/NF-κB/NLRP3/caspase-1/IL-1β signaling.	The association selenium-PDRN significantly improves morphological parameters in the testes, increases testosterone levels, and reduces NLRP3 inflammasome markers, indicating a positive effect on fertility.	[45]
PRP	Releases α-granules containing growth factors (PDGF, FGF, WNT) that stimulate cell proliferation and follicular regeneration.	Combined therapy with PRP and PDRN induces greater improvement in hair thickness in female pattern hair loss than PDRN therapy alone.	[40,41]
BMP2	Osteoinductive material for inducing differentiation of mesenchymal stems or progenitor cells.	Spontaneously forms a nanocomplex with PDRN via charge interaction, providing synergistic abilities in angiogenesis and bone regeneration while allowing for low doses of BMP2 to be used to avoid side effects.	[10,27,46]

**Table 4:** Summary of synergistic effects of PDRN with physical and electrical stimuli.

Stimulant	Primary Mechanism	Synergistic Role with PDRN	References
Microneedling	Mechanical Delivery and Micro-injury	Increases drug delivery efficacy by generating multiple microscopic channels and physical delivery channels; promotes tissue regeneration and wound repair while decreasing skin pigmentation via NNT modulation. Significantly maximizes penetration of PDRN in contracted nasal skin.	[9,24],
Laser Therapy	Thermal Energy and MTZ Remodeling	Improves wound healing and reduces recovery time after fractional laser resurfacing. Integration with LLLT significantly improves nerve recovery, nerve blood flow, and the restoration of nerve excitability/conduction by enhancing action potential thresholds.	[13,49]
ESWT	Mechanotransduction induces upregulation of growth factors (VEGF, IGF, FGF) and neovascularization via acoustic waves and cavitation stress.	Acoustic cavitation induces shearing stress and high pressure, facilitating PDRN delivery to target tissues and increasing A2AR binding. Enhances angiogenesis, collagen synthesis, and growth factor activation (VEGF, PECAM-1), leading to superior muscle regeneration and treatment of lateral epicondylitis.	[10,12,18,50,57]
Microcurrent Therapy	Bioelectrical Signaling	Mimics the bioelectrical strength of living tissue to increase ATP generation and VEGF release. Targets adenosine signaling in musculoskeletal disorders, enhancing chondrogenesis and promoting full-thickness rotator cuff healing through synergic regenerative mechanisms.	[15,52,56]
Pulsed Electromagnetic Fields	Upregulation of adenosine receptor density (A2AR and A3AR); non-invasive biophysical stimulation mimicking natural bone electrical activity.	Combined action targets A2ARs, increasing cAMP and activating PKA; a synergistic approach provides superior therapeutic results in inflammatory diseases such as osteoarthritis.	[15]
Bipolar Radiofrequency	Invasive microneedle electrodes deliver gated RF oscillations to induce wound repair and neocollagenesis.	Repetitive tissue wounding with minimal thermal coagulation maximizes PDRN utilization; the combination treatment remarkably softens contracted skin and improves mobility.	[47]

**Table 5:** Summary of therapeutic applications and clinical evidence of PDRN.

Stimulant	Primary Mechanism	Synergistic Role with PDRN	References
Mesenchymal stem cell-derived extracellular nanovesicles (NV), TI-EVs, or nSF-EPL-PDRN@Exo	A2A receptor activation and cAMP/PKA/CREB signaling pathway modulation	NV-DNA, TI-EVs, and exosomes synergistically promote angiogenesis, cellular proliferation, and ECM remodeling by activating complementary molecular pathways and facilitating the polarization of anti-inflammatory M2 macrophages.	[18,24,30]
L-carnitine, calcium ions, papain, or Arginine	Purinergic receptor stimulation, VEGF expression, and mitochondrial beta-oxidation	The combination acts synergistically to accelerate wound closure, increase wound-breaking strength, and increase epidermal markers such as collagen types I and IV compared to PDRN alone.	[22,39,47]
New Cellular Treatment Factor (NCTF)	A2A receptor activation, angiogenesis, and fibroblast activity regulation	NCTF provides a nutrient-rich milieu that enhances fibroblast proliferation and ECM synthesis, while PDRN promotes angiogenesis via VEGF upregulation; combined, they accelerate closure, epithelialization, and collagen organization.	[17]
Bacterial cellulose (BC) membrane	Topographic guidance (contact guidance) and A2A receptor activation	The aligned structure of BC guides cell migration and collagen orientation while PDRN enhances proliferation, reduces inflammation, and stimulates angiogenesis.	[31]

## Data Availability

No new data were created or analyzed in this study. Data sharing is not applicable to this article.
