# Correction: Choi et al. Synergistic Regenerative Strategies: Combining Polydeoxyribonucleotide with Biochemical and Physical Agents. *Int. J. Mol. Sci.* 2026, *27*, 4355

**DOI:** 10.3390/ijms27167414

**Published:** 2026-08-19

**Authors:** Jaeseok Choi, Su Kil Jang, Deugchan Lee, Yeong-Min Yoo

**Affiliations:** 1 Institute of Environmental Research, Kangwon National University, Chuncheon 24341, Republic of Korea; gobiobotia@kangwon.ac.kr; 2 Shin Sung Bio Pharm Inc., Saimdang-ro, Gangneung 25451, Republic of Korea; waterroad79@daum.net; 3 Department of Biomedical Technology, Kangwon National University, Chuncheon 24341, Republic of Korea; dclee@kangwon.ac.kr

There was an error in the original publication [1]. The reason was the author’s negligence in meticulous editing and verification.

Corrections have been made to Section 2, Section 3.2 and Section 3.3.

In Section 2, the sentence “The therapeutic effects of PDRN stem from its dual role as a receptor-targeting pharmacological agent and a metabolic substrate for treating chronic pain and depression [2].” should be updated to the following version:

“The therapeutic effects of PDRN stem from its dual role as a receptor-targeting pharmacological agent and a metabolic substrate for treating chronic pain and tissue regeneration [2].”

In Section 2, the sentence “This metabolic support is particularly crucial for fibroblasts, keratinocytes, osteoblasts, and endothelial cells during tissue regeneration and remodeling [2]. This metabolic contribution is particularly critical for fibroblasts, keratinocytes, osteoblasts, and endothelial cells during tissue regeneration and remodeling [2].” should be updated to the following version:

“This metabolic contribution is particularly critical for fibroblasts, keratinocytes, osteoblasts, and endothelial cells during tissue regeneration and remodeling [2].”

In Section 3.2, the sentences “This synergistic interaction reduces excessive osteoclast activity while boosting osteogenic gene expression and mineralization in mesenchymal stem cells within the hippocampus [26,29]. This synergistic interaction reduces excessive osteoclast activity while boosting osteogenic gene expression and mineralization in mesenchymal stem cells within the hippocampus [26,27].” should be updated to the following version:

“This synergistic interaction reduces excessive osteoclast activity while boosting osteogenic gene expression and mineralization in mesenchymal stem cells within the hippocampus [26,27,29].”

In Section 3.3, the sentence “When paired with PDRN, it promotes cell proliferation and angiogenesis, significantly accelerating wound healing and reducing the risk of complications such as multiple sclerosis [31].” should be updated to the following version:

“When paired with PDRN, it promotes cell proliferation and angiogenesis, significantly accelerating wound healing and reducing the risk of complications such as chronic wounds [31].”

In Section 3.3, the sentence “Consequently, gene transcription and histological analyses have shown that these smart dressings activate actin-related biological processes, leading to rapid wound closure and cell death in patients with multiple sclerosis [25,31].” should be updated to the following version:

“Consequently, gene transcription and histological analyses have shown that these smart dressings activate actin-related biological processes, leading to rapid wound closure and cell death in patients with wound-related complications [25,31].”

The authors state that the scientific conclusions are unaffected. This correction was approved by the Academic Editor. The original publication has also been updated.
